# Epidemiological Characteristics and Management of Multi-organ Donors in an Intensive Care Unit: An Eight-Year Retrospective Study

**DOI:** 10.7759/cureus.71556

**Published:** 2024-10-15

**Authors:** Luis Enrique Sanchez García, Nemi Isabel Pérez Peña, Francisco Aguilar Rodríguez, Daniela Esperanza Tolentino Pérez, Heaven Delhi Velarde Luján, José Manuel García Romero, Floricel O Villegas Amador, Alberto Montoya Vázquez, Jesus Adrian Diaz Ugalde, Irene García Hernández

**Affiliations:** 1 Transplant and Donation Department, Regional General Hospital 1 of the Mexican Social Security Institute, Queretaro, MEX; 2 Faculty of Medicine, Autonomous University of Queretaro, Queretaro, MEX

**Keywords:** brain death, donor maintenance, multi-organ donor, neurology and critical care, organ donation

## Abstract

Introduction: Patients with brain death are the primary source of organs for transplantation worldwide. Recognizing patients with this diagnosis, providing proper care, and effectively managing them in an intensive care unit (ICU) has been shown to increase donation rates in leading countries. The increasing demand for organs compared to the available number for transplantation heightens the responsibility of caring for potential donors.

Methods: An observational, cross-sectional, descriptive, and retrospective study was undertaken through the analysis of cases involving multiorgan donors diagnosed with brain death. This data was obtained from the coordination of donation and procurement at the Regional General Hospital 1 of the Mexican Institute of Social Security in Querétaro. The study encompassed the duration from January 1, 2016 to December 31, 2023.

Statistical analysis:A database was created in the Excel program and subsequently, the analysis of the variables identified was carried out in the IBM SPSS Statics 25 software (IBM Corp., Armonk, NY). Qualitative variables were analyzed using contingency tables, frequency tables, and statistical correlation tables. For quantitative variables, the analysis included averages, percentages, means, medians, modes, variances, and standard deviations.

Results: A total of 83 patients diagnosed with brain death were identified, of whom 56 became multiorgan donors. Thirty patients were excluded from donation, with the majority (19) due to family refusal. The primary age group was 10-20 years, accounting for 23.21% of cases. The mean age was 34.86 years. 71.43% of donors were male and 28.57% were female. The most frequent admission diagnosis was severe traumatic brain injury (46.43%). 56.6% of patients were admitted to the hospital with a Glasgow Coma Scale (GCS) score of 3 points. Brain death diagnosis was confirmed via angiography in 80.4% of cases. The average length of stay was 3.95 days. The average weight was 71.19 kg. The average height was 166 cm, and the mean BMI was 25.30. Forty patients had blood type O positive, accounting for 71.4%. A total of 255 organs and tissues were procured from 2016 to 2023: 103 kidneys, 97 corneas, 41 livers, six hearts, four skin and bone tissue derivatives, three pairs of lungs, and one heart valve. Hormone replacement therapy was not used in 55.4% of cases. Combined thyroid hormone and steroid regimen was used in nine patients (16.1%), and desmopressin was used in 12 patients (21.4%). During ICU stay, 69.6% of patients required norepinephrine to achieve perfusion goals.

Conclusions: The epidemiological profile characterizing the multiorgan donor at Regional Hospital 1 in Querétaro was typically male, with a mean age of 34.86 years, no comorbidities, presenting with severe traumatic brain injury upon arrival at the emergency department with a GCS score of 3 points. Norepinephrine was the most commonly used vasopressor, and the majority of patients did not meet hemodynamic criteria for initiating hormone replacement therapy.

## Introduction

Organ donation in Mexico is a generous, noble, and charitable deed. Currently, 20,115 recipients are waiting for a transplant, most of whom are seeking a kidney (16,617), cornea (3,245), liver (213), heart (23), liver-kidney (seven), kidney-pancreas (three), pancreas (three), heart-kidney (two), lung (one), and heart-lung (one), according to records from CENATRA (National Transplant Center) [1].

Despite the integration of doctors into donation coordination, the national donation rate in Mexico was 3.2 donations per million population (pmp) in 2007 and increased to 3.94 pmp in 2017 (a 23.1% increase) [2]. This ranks lower than countries with similar economies, governmental structures, and health care systems, including Argentina (13.4 pmp), Chile (9.6 pmp), Brazil (16.3 pmp), Colombia (8.9 pmp), Cuba (12.3 pmp), and Uruguay (18.9 pmp). Countries with different sociodemographic contexts greatly surpass these donation rates, such as the United States with 31.7 pmp, and Spain, the leader in donation, with 47 pmp [2].

In Mexico, health institutions have a hospital donation coordinator, and a physician responsible for coordinating procurement and transplant activities. It is essential to identify potential donors by analyzing hospital deaths according to age, contraindications, causes of death, etcetera, to determine if they are potential tissue donors (in the case of patients who die from cardiopulmonary arrest) and organ donors (brain death). They then work to raise awareness among families and request their permission to proceed with organ procurement, a practice recommended since 1998. This is supported by higher preservation rates when authorization requests are submitted by professionals possessing more experience and a genuine commitment to helping families understand and navigate through the organ donation process [3].

The identification of patients who may potentially progress to brain death is a current challenge for donation coordinators. CENATRA recommends monitoring and initiating protocols for patients with a Glasgow coma scale (GCS) below 7. As a leader in donation, Spain has established several recommendations and criteria proposed by the National Protocol for the (Maintenance of Potential Organ Donors (SEMICYUC-ONT), which considers variables that predict progression to brain death: abolition of three brainstem reflexes upon arrival in the emergency department and certain tomographic criteria such as hematoma volumes in brain hemorrhages (greater than 65 cc), presence of a swirl sign on non-contrast computed tomography (active bleeding), as well as midline shift and effacement of cisterns [4].

Diagnosis of brain death

Brain death is defined as the irreversible loss, due to a known cause, of the functions of all intracranial neurological structures, including cerebral hemispheres and the brainstem. The most common diagnoses that progress to this condition are severe traumatic brain injury (40-50%), hemorrhagic stroke (30-35%), and less frequently, intracerebral hemorrhage (epidural, subdural, parenchymal), hypoxic-ischemic encephalopathy, ischemic stroke, and intracranial tumors [5].

The Mexican General Health Law establishes that the diagnosis of brain death must be confirmed through imaging studies (Article 344 [5]). An Electroencephalogram showing a total absence of electrical activity, confirmed by a specialist physician or any other imaging study that documents the permanent absence of arterial cerebral flow-like brain angiotomography.

The ICU is essential for the processes of donation, donor maintenance, and the diagnosis of brain death, as well as perioperative and postoperative care in hospitals licensed to transplant organs [6]. The growing context of organ demand, compared to the number of available organs for transplantation, increases the responsibility of caring for potential donors.

## Materials and methods

An observational, cross-sectional, descriptive, and retrospective study was undertaken through the analysis of cases involving multiorgan donors diagnosed with brain death. The data were obtained from the coordination of donation and procurement at the Regional General Hospital 1 of the Mexican Institute of Social Security in Querétaro. The study encompassed the duration from January 1, 2016 to December 31, 2023. The inclusion criteria are delineated as follows: individuals aged over two years and under 70 years, who have been diagnosed with brain death through a study conducted within the medical institution (such as cerebral angiotomography or electroencephalography) interpreted by certified radiologists or neurologists, in conjunction with the patient's medical history. Exclusion criteria included patients under two years and over 70 years, patients diagnosed with cancer, sepsis, history of autoimmune diseases, corneal injuries, and corneal surgery. The criteria for elimination included: incomplete records and variables that were unavailable due to the loss of records.

The study used convenience sampling. We included all multi-organ donations during the study period that met eligibility criteria and obtained informed consent from donor families. This approach was based on the availability of eligible cases during the study period. No random or probability sampling technique was employed.

**Figure 1:**
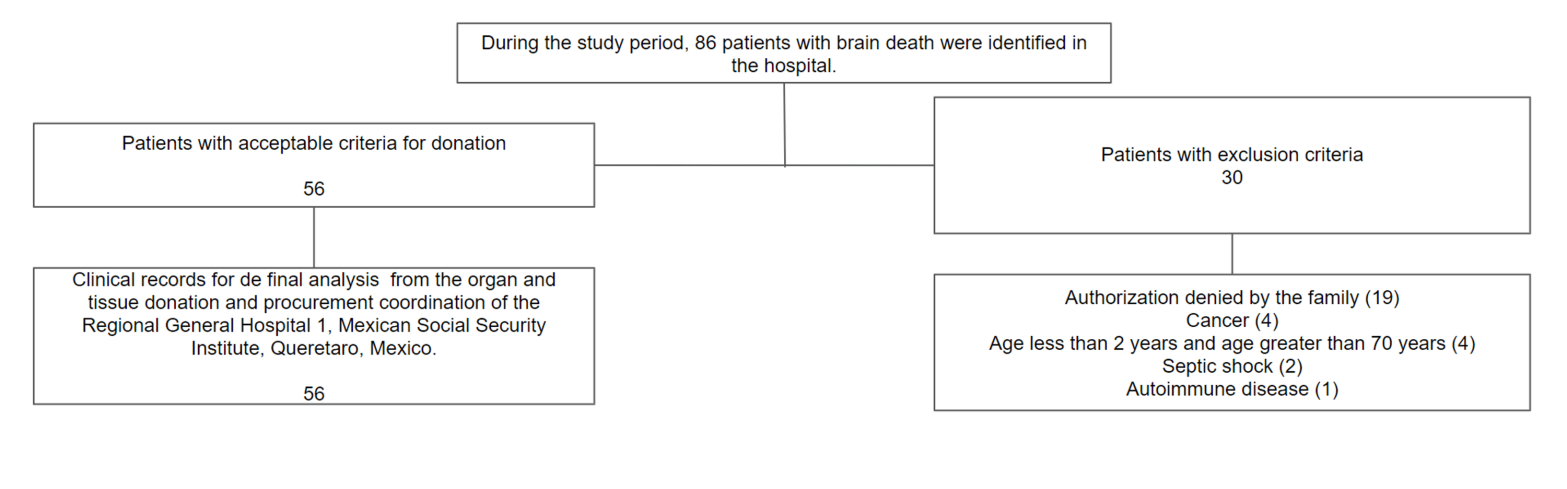
Flowchart of selection criteria for final study sample The number of patients selected as well as those excluded from the study on the eligibility criteria for multiorganic donation are shown. Additionally, incomplete and inconsistent medical records were excluded.

The physical and electronic records of the patients included in the study were analyzed. A database was created in the Excel program and subsequently, the analysis of the variables identified was carried out in the IBM SPSS Statics 25 software (IBM Corp., Armonk, NY). Epidemiological data such as age, gender, weight, height, BMI, blood hemotype, cause of hospitalization, comorbidities described in the admission medical history, Glasgow on arrival, imaging techniques for diagnosing brain death (cerebral angiotomography or electroencephalogram), number of organs procured, patient complications, hormone replacement therapy used in each patient, and hemodynamic parameters such as blood pressure, hemodynamic parameters such as mean arterial blood pressure, respiratory and gasometric parameters (Positive end-expiratory pressure, ph scale and arterial pressure of oxygen and carbon dioxide). Laboratories obtained through the hospital platform include hemoglobin levels, platelet, and leukocyte counts, coagulation times, electrolytes, creatinine measurements at the time of hospital admission, creatinine levels upon admission to intensive care, and creatinine values prior to organ procurement, as well as plasma glucose levels and the urinary output index. Qualitative variables were analyzed using contingency tables, frequency tables, and statistical correlation tables. For quantitative variables, the analysis included averages, percentages, means, medians, modes, variances, and standard deviations (Figure 1).

The protocol was approved by the donor families in addition to the research committee (Institutional Registration R-2024-2201-117) and bioethics (Registration COFEPRIS Federal Commission for Protection against Health Risks 20 CI 22 014 028 CONBIOETICA 22 CEI 001 2018073).

## Results

From January 2016 to December 2023, 86 patients were identified with a brain death diagnosis, and their management was conducted in accordance with the guidelines established by CENATRA. A total of 56 patients were identified as multi-organ donors, whereas 30 patients were deemed ineligible for donation. The reasons for exclusion included family refusal in 19 cases, metastasis in four instances, classification as end-of-life cases in another four, established septic shock in two patients, and one patient who was excluded due to an autoimmune disease.

**Figure 2:**
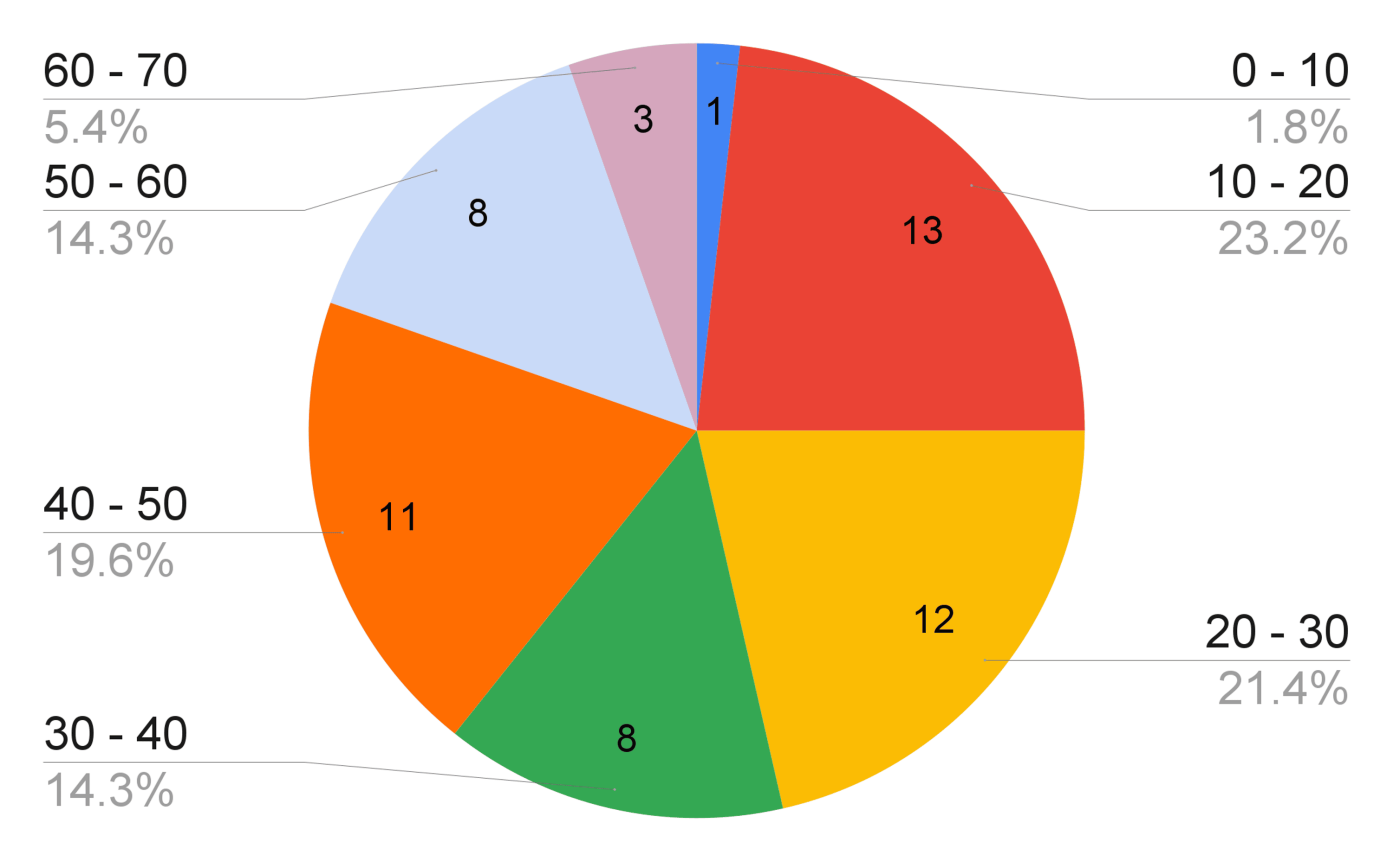
Age of multi-organ donors during the study period

A total of 56 multi-organ donations. The age ranges found were as follows: 0 to 10 years 1.79% (one patient), 10 to 20 years 23.21% (13 patients), which was the age group with the highest number of multi-organ donors; 20 to 30 years 21.43% (12 patients); 30 to 40 years 14.29% (eight patients); 40 to 50 years 19.54% (11 patients); 50 to 60 years 14.29% (eight patients); and finally, the range of 60 to 70 years with 5.36% (three patients) (Figure 2).

The average donor’s age was 34.86 years. The youngest patient was a nine-year-old pediatric patient, and the oldest donor was 66 years. Among the 56 multi-organ donor patients, 71.43% were male (40 donors) and 28.57% were female (16 donors). Furthermore, 55.36% of these individuals succumbed to causes necessitating notification to the public prosecutor's office (31 patients), whereas the remaining 44.64% passed away from causes that did not require such notification (25 patients).

Regarding the donor's weight, we found an average of 71.19 kg, with the lowest recorded weight being 28 kg and the maximum 120 kg. The average height was 1.66 m, with a minimum height of 1.32 m and a maximum of 1.90 m. In terms of Body Mass Index (BMI), we found an average of 25.30, with the minimum reported BMI being 15.36 and the maximum 33.24. We identified 44.6% of donors with a normal or adequate weight BMI, 41.1% with overweight, and 14.3% with obesity (Table 1). Of the 56 donors, 40 patients had O positive blood type, representing 71.4%, 13 patients had A positive (23.2%), two patients had B positive (3.6%), and one patient had AB positive (1.8%).

**Table 1:** Epidemiological characteristics of the multiorgan donor BMI: Body mass index

Characteristics	Total (N=56)
Age	34.86 years old (average)
Medical or legal case	Medical: 44.64%, Legal case: 55.36%
Weight	71.19 kg (average)
BMI	25.30 (average)
Normal BMI	44.6%
Overweight BMI	41.1%
Obesity BMI	14.3%
Gender	71.43% Men, 28.57% Women
Blood type	
O	71.4%
A	23.2%
B	3.6%
AB	1.8%
Comorbidities	
No comorbidities	73.2%
Hypertension	12.5%
Type 2 diabetes	10.7%
Days of hospital stay	3.95 days
Patients with a Glasgow Coma Scale 3	30 patients (53.6%)
Method used to diagnose brain death	Angiotomography: 80.4%, Electroencephalogram: 19.6%

**Figure 3:**
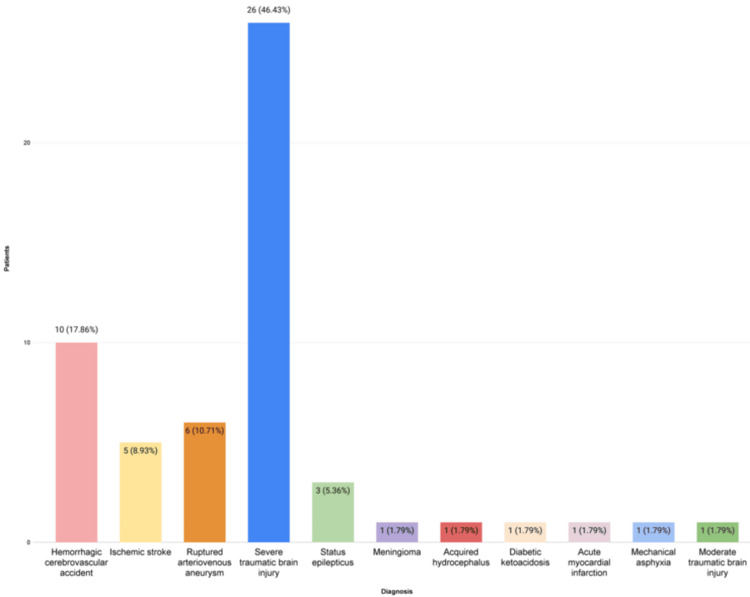
Causes of hospitalization of multi-organ donors during the study period

The causes of hospitalization for the donor patients were analyzed, finding that severe traumatic brain injury was the primary admission diagnosis, accounting for 46.43% (26 patients), followed by hemorrhagic stroke at 17.9% (10 patients), and in third place, the cerebral arteriovenous aneurysm rupture at 10.7% (six patients). In fourth place was ischemic stroke at 8.9% (five patients), followed by status epilepticus at 5.4% (three patients). Meningioma, acquired hydrocephalus, diabetic ketoacidosis, acute myocardial infarction, mechanical asphyxia, and moderate traumatic brain injury each accounted for 1.8% (one patient each for these conditions) (Figure 3).

**Figure 4:**
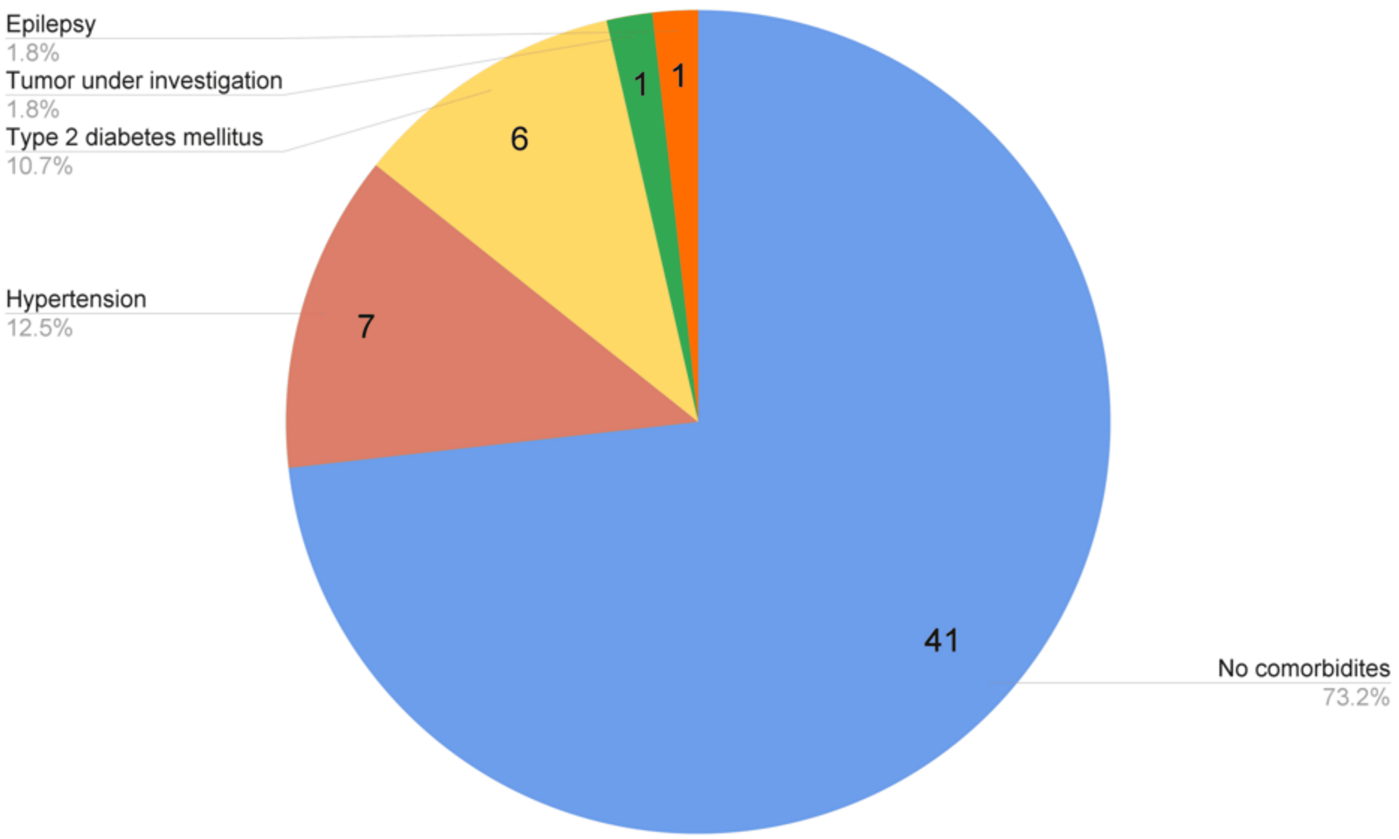
Comorbidities of multi-organ donors during the study period

The majority of patients had no comorbidities, accounting for 73.2% (41 patients). The main comorbidity detected was systemic arterial hypertension at 12.5% (seven patients), followed by type 2 diabetes at 10.7% (six patients). One patient presented with a tumor under study (meningioma) and another one with epilepsy, each one representing 1.8% (Figure 4).

Thirty patients presented with a GCS score of 3 upon admission, representing 53.6%. Seven patients had a GCS score of 6 points (12.5%), six patients had 8 points (10.7%), and three patients (5.4%) had a GCS score of 4. Three patients had a score of 13, also representing 5.4%. Two patients presented with 10 points, and two patients had a GCS score of 15, representing 3.6%. Only three patients were admitted with GCS scores of 7, 9, and 11, representing 1.8%.

Forty-five patients were diagnosed with brain death through an angiography, representing 80.4%, while 11 patients were diagnosed through an electroencephalogram (19.6%). We found an average hospital stay of 3.95 days, with a minimum of one day and a maximum of 19 days.

**Figure 5:**
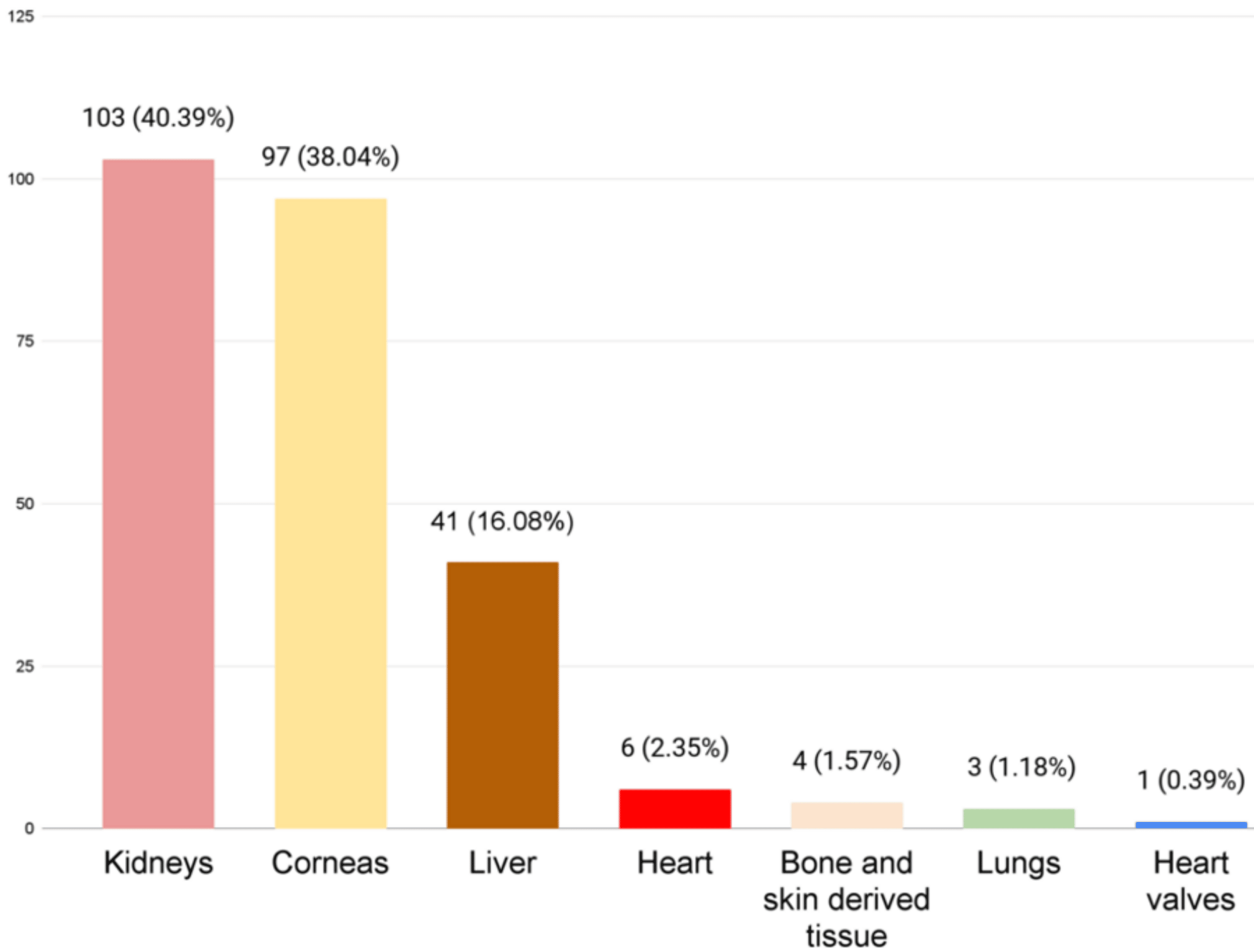
Total organs donated during the study period

Of the 56 donors, 26 had five organs procured, representing 46.4%; nine patients had three organs (16.1%); eight patients had four organs (14.3%); five patients had six organs (8.9%); three patients had two organs; and another three patients had seven organs (5.4% each). One patient had one organ, and another had eight organs, representing 1.8%. A total of 255 organs and tissues were procured from 2016 to 2023, divided as follows: 103 kidneys, 97 corneas, 41 livers, six hearts, four tissues derived from skin and bone, three pairs of lungs, and one heart valve (Figure 5).

Most of our patients (46.43%) had no complications during their stay (26 patients). A total of 19.54% were diagnosed with acute kidney injury (11 patients), 19.6% with diabetes insipidus (11 patients), 10.7% with hypernatremia (six patients), and 3.6% with hypokalemia (two patients).

It was documented that in 31 patients, representing 55.4% no measures for hormone replacement therapy were employed. Additionally, the concomitant administration of thyroid hormones alongside a steroid regimen was observed in nine patients. (16.1%), 12 patients were managed using desmopressin (21.4%), exclusive use of thyroid hormone was reported in one patient (1.8%), and three patients needed use of desmopressin, thyroid hormone, and steroid, representing 5.4% (Table 2).

**Table 2:** Pharmacological management of the multiorgan donor in the intensive care unit Corticosteroids including methylprednisolone, prednisolone, hydrocortisone, or dexamethasone

Vasopressor medication	
No vasopresor medication required	5.4%
Norepinephrine	69.6% (39 patients)
Dopamine	14.3% (8 patients)
Vasopressin	10.7% (6 patients)
Use of hormonal replacement in the multiorgan donor	
Did not use hormonal replacement therapy	55.4% (31 patients)
Thyroid hormone + Corticosteoid	16.1% (9 patients)
Intranasal desmopressin	21.4% (12 patients)
Thyroid hormone	1.8% (1 patients)
Corticosteroid	5.4% (3 patients)

Within the obtained hemodynamic parameters, the average mean arterial blood pressure of the donors' prior multi-organ procurement was 80.57 mmHg, with a minimum of 56 mmHg and a maximum of 100 mmHg. All of our donors had central venous access during their stay in the ICU 5.4% of the patients (three) did not require vasopressors to maintain hemodynamic targets. 69.6% used norepinephrine (39 patients), 14.3% used dopamine (eight patients), and 10.7% used vasopressin (six patients).

Regarding the ventilatory parameters documented in previous multi-organ procurement, we found that 100% of the patients were connected to a volume-controlled ventilation mode. The documented blood gas analyses nearest to the time of procurement showed an average arterial carbon dioxide pressure of 36.50 mmHg, with a minimum of 26 mmHg and a maximum of 56 mmHg. The average pH was 7.39, with a minimum of 7.10 and a maximum of 7.56. The mean tidal volume was 414 mL. The mean PEEP was 5.42, with a median of 5; the minimum found was 3 cmH2O and the maximum was 10 cmH2O. 89.3% were within target levels and 10.7% were not. The FiO2 (fraction of inspired oxygen) ranged from 21% to 80%, with an average of 43.14% (Table 3).

**Table 3:** Clinical and laboratory data of multiorgan donor patients MAP: Mean arterial pressure, PEEP: Positive and expiratory pressure, ICU: Intensive care unit

Hemodynamic parameters	
Mean arterial pressure (MAP)	80.57 mmHg (Average)
Ventilatory parameters	
Volume-controlled ventilation	100%
Positive and expiratory pressure PEEP	5.42 cmH2O (Average) 5 median
PEEP within goals	89.3%
PEEP outside goals	10.7%
Fraction of inspired oxygen	43.14% (Average)
Arterial carbon dioxide CO2 before procurement	36.50 mmHg (Average)
Arterial pH before procurement	7.39 pH (Average)
Blood count	
White blood cells	13.86 miles/µL (Average)
Hemoglobin	12.81 g/dL (Average)
Hemoglobin within goals	87.5%
Hemoglobin outside goals	12.5%
Platelets	161,857/mm3
Platelets within goals	92.9%
Platelets outside goals	7.1%
Urinary output	1.26 mL/kg/h
Urinary output (>1 mL/kg/h) within goals	85.7%
Urinary output outside goals	14.3%
Creatinine	
Creatinine at emergency department admission	1.46 mg/dL (Average)
Creatinine at ICU admission	0.40 mg/dL (Average)
Creatinine before multiorgan procurement	0.50 mg/dL (Average)
Glucose	159.82 mg/dL (Average)
Glucose within goals (<180 mg/dL)	76.8%
Glucose outside goals	23.2%
Electrolytes	
Phosphorus	3.9 mg/dL (Average)
Calcium	8.3 mg/dL (Average)
Chloride	114.23 mEq/L (Average)
Potassium	4.19 mEq/L (Average)
Sodium	147.55 mEq/L (Average)
Magnesium	1.85 mg/dL (Average)

The latest results for the coagulation tests before multi-organ procurement showed an average prothrombin time of 16.20 seconds, with a minimum of 11 seconds and a maximum of 26.30 seconds. According to these results, 51.8% were within target levels and 48.2% were not. The partial thromboplastin time had an average of 32 seconds, with a minimum of 16.40 seconds and a maximum of 55.60 seconds, reaching target levels of 80.4% and outside target levels of 19.6%. Finally, the international normalized ratio was reported with an average of 1.35, a minimum of 0.90, and a maximum of 2.11, with 92.9% within target levels and 7.1% outside target levels.

The latest complete blood count before procurement included an analysis of the white blood cell series, showing an average of 13.86 thousand/µL, with a minimum of 5.10 thousand/µL and a maximum of 27.90 thousand/µL. For hemoglobin levels, we observed an average of 12.81 g/dL, with a minimum of 7.10 g/dL and a maximum of 21.50 g/dL, achieving 87.5% within target levels and 12.5% outside of targets. The average hematocrit was 39.44%, with a minimum of 19.43% and a maximum of 65.10%. Finally, the average platelet count was 161,857/µL, with a minimum of 26,000/µL and a maximum of 381,000/µL, resulting in 92.9% within target levels and 7.1% outside of targets.

In relation to nephrological data, we documented an average urinary output index of 1.26 mL/kg/h among our donors prior to procurement. Consequently, 85.7% of them achieved target levels of > 1 mL/kg/h, while 14.3% did not.

Creatinine levels were measured at three different times. The first measurement was taken upon the patient's admission to the emergency department (which were their initial laboratory tests), revealing an average of 1.46 mg/dL, with a minimum of 0.30 mg/dL and a maximum of 9.84 mg/dL. Subsequently, upon admission to the ICU, the average was 0.40 mg/dL, with a minimum of 0.40 mg/dL and a maximum of 7.80 mg/dL. Finally, the last creatinine measurement before organ procurement had an average of 1.35 mg/dL, with a minimum of 0.50 mg/dL and a maximum of 5.40 mg/dL.

Central glucose levels were measured before procurement, finding an average of 159.82 mg/dL, with a minimum of 70 mg/dL and a maximum of 321 mg/dL. Our patients were within target levels in 76.8% of cases and outside target levels in 23.2%.

Finally, we analyzed the serum electrolytes closest to the time of procurement, finding the following averages: phosphorus at 3.9 mg/dL, calcium at 8.3 mg/dL, chloride at 114.23 mEq/L, potassium at 4.19 mEq/L, sodium at 147.55 mEq/L, and magnesium at 1.85 mg/dL.

## Discussion

As of today, both locally and globally, there is limited information on statistics regarding brain death, epidemiological data on multi-organ donors, and the management of patients in Mexican ICUs at the second and third levels. The diagnosis of brain death is presently subject to rigorous regulation by medical institutions, however many terms associated with this condition remain unfamiliar to healthcare professionals. A study published in 2016 by the Medical Journal of the Mexican Social Security Institute shows that a high percentage of healthcare staff have partial knowledge of the law regarding brain death and its clinical concepts; 68% of the surveyed population is unaware of the complementary studies established to confirm the diagnosis of brain death [7]. The presentation of analyzed data leads to a better understanding of donors in retrospect, including age, gender, causes of hospital admission, and the management provided by different healthcare units.

Among the potential limitations of the research, we find the reliance on medical evolution notes obtained from the PHEDS platform and physical files, as this is a retrospective study. An area for enhancement in future related investigations would be the implementation of a prospective study, similar to the DONORS study in Brazil [8].

During the study period, we collected information on 83 patients for whom a diagnosis of brain death was established, by Article 343 of the General Health Law. A limitation of our study is the lack of total death records per year, which would allow us to determine the frequency of brain death diagnoses in our hospital. A study conducted in European intensive care units included 4,248 deceased patients, of whom 330 (7.8%) died from brain death [6]. In our country, there are few studies on this topic; one of them reports that 10.21% of admissions to intensive care units met the criteria for potential donors, which is similar to data from the European Union [9]. 

Of the 83 patients diagnosed with brain death, 30 were excluded due to family refusal. These data align with Fortuna (2014), which reported a 22.14% rate of cases eliminated due to family refusal, and another study from 2017 that reported a family refusal rate of 23% [10]. Of the 83 patients diagnosed with brain death, 56 were able to become multi-organ donors. The age range with the highest number of donors was 10 to 20 years, representing 23.21%, with an average age of 34.86. These data exhibit divergence from statistics reported by the European Union, wherein the average age of adult donors increased from 38 years in 1992 to 60.7 years in 2013 [4] At the same time, a study conducted in Seoul in 2023 reports an average age of 52 years [11]. On the other hand, Fortuna observed an average age of 36.08 years, which is very close to our results. 71.43% were male donors and 28.57% were female. These figures align with international research regarding the gender of donors and recipients, which shows that the predominant source of donations is deceased males, accounting for 64.3% across 64 countries with a total record of 39,983 patients [12].

Among the causes of hospitalization of patients, we found severe traumatic brain injury as the primary diagnosis, accounting for 46.43%, followed by a hemorrhagic stroke at 17.9%, and in third place, arteriovenous aneurysm rupture at 10.7%. According to data from the European Union, in Spain, the main cause of brain death in donors is intracerebral hemorrhage at 42%, followed by TBI at 19% [4]. The study led by Lee in 2023 is the most closely aligned with our methodology. Among the main causes of death, cerebral hemorrhage was reported in 61.5% of cases, divided into intracerebral (39%), subarachnoid (39.6%), and subdural (20.8%) hemorrhages [11]. These latest data, together with our findings, are both interesting and consistent with international literature, which highlights that severe subarachnoid hemorrhage related to aneurysm rupture is often associated with poor prognosis and a high probability of progression to brain death [4,6]. Among our donors, the majority had no comorbidities (73.2%). The most common comorbidity detected was systemic arterial hypertension at 12.5%, followed by type 2 diabetes mellitus at 10.7%. This is similar to what was reported by Lee in 2023, with hypertension present in 30.4% of their donors and diabetes in 10.1% [11].

Most of our patients were admitted with a GCS score of 3 points, representing 53.6% of the cases. According to SEMICYUC, a score below 6 is considered a determining factor that increases the probability of progression to brain death [4]. Our donors had an average hospital stay of 3.95 days, which is slightly different from the data reported by Lee in 2023, where the median ICU stay was 13.62 days [11]. Nevertheless, this is consistent with data presented by Fortuna in 2014, where the time from admission to the ICU was found to be an average of 21.01 hours [9].

Regarding the anthropometric data pertaining to our donors, we found an average weight of 71.19 kg, an average height of 1.66 m, and an average BMI of 25.30. Most of our donors (44.6%) had a normal BMI or adequate weight according to the WHO standards. This differs from the data reported in Seoul, where the majority of their patients had a BMI of 28.18 [11].

Out of the 56 donors, 40 patients had an O-positive blood type, representing 71.4%, 13 patients had A positive (23.2%), two patients had B positive (3.6%), and one patient had AB positive (1.8%). This is consistent with reports from other countries where blood type O is the most common (29%) and AB is the least frequent [12].

Out of the 56 donors, 26 were procured for five organs, representing 46.4%. A total of 255 organs and tissues were procured during the period from 2016 to 2023, divided as follows: 103 kidneys, 97 corneas, 41 livers, six hearts, four tissues derived from skin and bone, three pairs of lungs, and one cardiac valve. Fortuna reports a total of 57 organs, divided into 12 livers, 44 kidneys, and one heart; however, this study has a shorter observation period than ours [9]. It is important to highlight the positivity rate of corneal tissue donors at 8.6% in the pre-procurement analyses (viral panel) detected by Peña in the same sample population but over a shorter study period [13].

The majority of our patients (26), representing 46.43%, did not experience any complications during their stay. However, 19.54% were diagnosed with acute kidney injury (11 patients), 19.6% with diabetes insipidus (11 patients), 10.7% with hypernatremia (six patients), and 3.6% with hypokalemia (two patients). International literature recognizes diabetes insipidus as the primary endocrinopathy related to brain death [14], reporting rates between 46% and 86%.

It was documented that 55.4% of our patients did not receive hormonal replacement therapy. Despite the theoretical benefits of hormonal replacement therapies, primarily with thyroid hormone and systemic steroids, international guidelines and the majority of literature found in our research only provide recommendations regarding the implementation of this management. Corticosteroids and thyroid hormone replacement have not demonstrated any benefit in patients hemodynamically stable [15]; their use is solely recommended for those exhibiting hemodynamic instability who have already been managed with fluids and vasopressors.

In our study, 69.6% of patients required norepinephrine to achieve hemodynamic targets. This is the primary vasopressor recommended according to the Mexican CENATRA guidelines [5]. Nevertheless, international literature remains inconsistent; studies from Ireland in 2018 recommend vasopressin as a first-line agent [6]. While another study suggests dopamine as a first-line vasopressor [14].

Our patients had an average mean blood arterial pressure of 80.57 mmHg, which aligns with the majority of recommendations provided by intensive care societies with targets of >65 mmHg, 70-80 mmHg, and >70 mmHg, respectively, aiming to maintain euvolemia, reduce the use of vasoconstrictors, and improve cardiac output.

Regarding respiratory management and the tendency of patients with brain death to develop neurogenic pulmonary edema, we found that 100% of our patients were on a volume-controlled ventilation mode. They were managed with protective ventilation and a PEEP with an average of 5.42, while international literature recommends a PEEP between 5 and 10 [14,16]. Our patients reported an average FiO_2_ of 42.14%. The evidence on this topic is diverse, but it is generally recommended to maintain a FiO2 that achieves a saturation between 94% and 95% [15,17]. The CENATRA guidelines ideally recommend maintaining the FiO2 below 40%. Regarding total volume, our donors reported an average of 414 mL, while protective ventilation with volumes between 6 and 10 mL/kg of ideal body weight is recommended [14,16].

We obtained the latest measurements before multiorgan procurement regarding coagulation times. We found that the prothrombin time was within target in 51.8% of cases, and the partial thromboplastin time was within target in 80.4%. In general, it is recommended to achieve acceptable values to prevent bleeding. Additionally, the latest pre-procurement measurements of the complete blood count showed an average white blood cell count of 13.86 × 10⁹/L and an average hemoglobin level of 12.81 mg/dL, with 87.5% within target. According to most intensive care societies, the recommended target is above 7 mg/dL in stable donors and 9 mg/dL in cases of hemodynamic instability [5,14]. The recommendations regarding platelet levels are >50,000/µL.14 y > 80,000/µL [17]. Regarding our patients, we had an average platelet count of 161,000/µL.

The diuresis targets align with the majority of international recommendations [14,16]. Our patients met the proposed targets in 85.7% of cases, maintaining an average diuresis of 1.26 mL/kg/h. Despite the absence of well-established targets for creatinine levels, and acknowledging the limitations in determining the glomerular filtration rate-such as the fact that creatinine is not reabsorbed but is secreted by the proximal tubule and the gut microbiome can degrade it, and other factors like muscle mass, protein intake, and exercise [18], it remains our primary marker for detecting acute kidney injury and approximating the glomerular filtration rate in our patients. We established three measurement points: the initial one upon admission, where our patients had an average creatinine level of 1.46 mg/dL followed by a second measurement upon ICU admission, with an average of 1.75 mg/dL; and the last average creatinine level was noted as 1.35 mg/dL.

Regarding glycemic management in patients with brain death, it is generally recommended to keep glucose levels below 180 mg/dL [14,16]. Our patients had an average glucose level of 159.82 mg/dL, meeting the proposed targets in 76.8% of cases.

## Conclusions

It is concluded that the epidemiological profile characterizing the multiorgan donor from the Regional Hospital 1 in Querétaro was a male donor, with an average age of 34.86 years, without comorbidities, and diagnosed with severe traumatic brain injury, who entered the emergency department with a GCS score of 3. Norepinephrine is established as the most commonly used vasopressor in our hospital unit. The majority of our patients did not meet the hemodynamic criteria to initiate hormonal replacement therapy with thyroid hormone and steroids.

As a second-level medical unit licensed for tissue and organ procurement, part of the donation coordination activities includes spreading information regarding various aspects, including the sociodemographic characteristics of our donors and the care they receive within the ICU, emphasizing the fundamental role played by the hospital coordinator and the intensivist in enhancing donation rates at both local and national levels.
